# Tumor immune microenvironment states inferred from TLS-associated immune-cell composition stratify prognosis in hepatocellular carcinoma

**DOI:** 10.3389/fimmu.2026.1818138

**Published:** 2026-05-28

**Authors:** Chihyuan Cheng, Geng Chen, Jing Zhang, Liman Qiu, Zhenli Li, Xiuqing Dong, Chenfan Lu, Qiming Wu, Xiaohui Peng, Zhixiong Cai, Yongyi Zeng

**Affiliations:** 1 The First Affiliated Hospital of Fujian Medical University, Fuzhou, China; 2 The Fifth Hospital of Xiamen, Xiamen, China; 3 The United Innovation of Mengchao Hepatobiliary Technology Key Laboratory of Fujian Province and Mengchao Hepatobiliary Hospital of Fujian Medical University, Fuzhou, China

**Keywords:** hepatocellular carcinoma, immune-cell composition, immunotherapy response, tertiary lymphoid structures, tumor immune microenvironment

## Abstract

**Background:**

Tertiary lymphoid structures (TLS) are ectopic immune hubs in the tumor immune microenvironment (TIME) associated with prognosis and immunotherapy response, yet commonly used TLS structural descriptors show inconsistent associations with prognosis in hepatocellular carcinoma (HCC). Here, we propose a TLS-associated immune-cell composition framework that quantifies the TIME state and predicts patient outcomes.

**Methods:**

By integrating an HCC single-cell atlas with literature-curated TLS gene sets, we defined six TLS-associated immune components (TLS6). Using TLS6 as a reference, we applied BayesPrism deconvolution to infer the relative abundance of TLS6 from bulk tumor transcriptomes. Given the divergent prognostic associations across TLS6 fractions, we applied LASSO–Cox regression to derive a two-feature TLS RiskScore retaining regulatory T cells and cDC2 cells.

**Results:**

The TLS RiskScore stratified overall survival in TCGA-LIHC and ICGC LIRI-JP and was associated with response to PD-1 blockade in an independent anti–PD-1–treated HCC cohort. In multicenter FFPE tissues, a multiplex immunofluorescence (mIF) implementation quantifying TLS-localized CD4^+^FOXP3^+^ regulatory T cells and CD11c^+^CD1c^+^ cDC2 cells reproduced prognostic stratification without model refitting.

**Conclusions:**

Collectively, these results support a compact, translatable TLS-associated immune-cell composition framework that provides a computable TIME-state measure associated with prognosis and response to PD-1 blockade in HCC.

## Introduction

Hepatocellular carcinoma (HCC) is characterized by profound substantial molecular, cellular, and spatial heterogeneity (1), as well as effective immune evasion, which together contribute to a highly immunosuppressive tumor immune microenvironment (TIME) (2, 3). Targeting this immunosuppressive TIME has emerged as a key therapeutic strategy (4), and the incorporation of immune checkpoint inhibitor (ICI)–based combinations into first-line systemic therapy has improved outcomes in a subset of patients (1, 2). However, overall survival remains suboptimal, and clinical outcomes vary widely (2, 5), highlighting the need for robust biomarkers that accurately capture HCC TIME states to inform prognosis and predict immunotherapy response (2).

Tertiary lymphoid structures (TLS) serve as ectopic immune hubs within the TIME, where they facilitate antigen presentation and lymphocyte activation, and are therefore often considered indicators of local immune organization (3, 4, 6). Studies across multiple solid tumors have shown that the presence and maturation of TLS are associated with favorable prognosis and improved responses to immunotherapy (6–8). In HCC, however, these associations remain inconsistent: whereas some studies report prolonged survival, others have found that TLS maturation is associated with early recurrence risk in HCC (4, 5, 9). Early studies focused mainly on structural features—TLS presence, density, maturation, and spatial distribution (4, 6, 10). Although descriptive, these structural measures cannot account for differences in the immune-cell composition and immune programs of structurally similar TLS (6, 11). As TLS biology became better understood, subsequent work shifted toward quantifying selected TLS-associated immune cell populations using immunohistochemistry, multiplex imaging, or bulk transcriptomic signatures (8, 11–13). However, these approaches remain centered on individual cell types and therefore fail to capture how multiple TLS-associated components jointly define TLS immune programs (6, 12).

To address this limitation, we adopt a more comprehensive view of TLS as components of the tumor immune microenvironment (TIME), considering both their immune-cell composition and the immune programs of constituent cells. We then asked whether variation in TLS composition and associated programs is linked to clinically relevant microenvironmental states, prognosis, and response to immunotherapy in HCC. To this end, we integrated literature-derived TLS gene modules with a published single-cell atlas of the HCC TIME (14) to define TLS-associated immune components and derive a compact TLS RiskScore from bulk tumor transcriptomes. Across independent cohorts and an orthogonal multiplex immunofluorescence (mIF) implementation in FFPE tissues, the score was associated with survival and clinical benefit from PD-1 blockade, supporting its utility as a computable TIME-state measure for risk stratification in HCC. Collectively, these analyses demonstrate that the TLS RiskScore links TLS-associated immune patterns to survival and benefit from PD-1 blockade in HCC, supporting its use for prognostic modeling and immunotherapy stratification.

## Materials and methods

### Human samples and ethics

Formalin-fixed, paraffin-embedded (FFPE) tissue specimens were obtained from patients with hepatocellular carcinoma (HCC) who underwent surgical resection at Mengchao Hepatobiliary Hospital, Fujian Medical University, and the Fifth Hospital of Xiamen between 2017 and 2023. The specimens were derived from archived surgical pathology FFPE tissue blocks.

At Mengchao Hepatobiliary Hospital, this retrospective study used biospecimens and associated clinicopathologic data from the institutional biobank, which was approved by the Medical Ethics Committee of Mengchao Hepatobiliary Hospital, Fujian Medical University (Ethics approval No. AF/SW-07/02.0). Written informed consent for biobank storage and research use was obtained from all participants at sample collection. Access to biobank materials was granted through the biobank’s established governance and ethics review procedures; therefore, re-consent was not required for this retrospective analysis. At the Fifth Hospital of Xiamen, retrospective use of archival FFPE tissues and related data was approved by the Medical Ethics Committee of the Fifth Hospital of Xiamen (Approval No. XMWY-KY-2025-051). All specimens and associated clinicopathologic data were de-identified before release for research and before analysis.

Using a two-stage selection process, we screened 113 consecutive resected HCC cases for eligibility and tissue adequacy according to prespecified criteria. Of the screened cases, 36 that met the prespecified criteria were selected for multiplex immunofluorescence (mIF) validation.

### Public data resources

Publicly available datasets analyzed in this study were retrieved from established cancer genomics repositories (GEO, the TCGA/GDC portal, and the ICGC data portal), including scRNA-seq data (GEO accession GSE149614; paired tumor and adjacent liver samples) (14), bulk RNA-seq data for model training (TCGA-LIHC), bulk RNA-seq data for external validation (ICGC LIRI-JP), a bulk RNA-seq anti–PD-1–treated HCC cohort (GEO accession GSE202069) (15), and processed spatial transcriptomics (ST) data for HCC (deposited to Mendeley Data, dataset ID skrx2fz79n) (16).

### Single-cell RNA sequencing and preprocessing

Raw UMI count matrices from 10x Genomics Chromium scRNA-seq data (GEO accession GSE149614) were processed using Seurat (v5.0.0) (17) in R (v4.3.0). Quality control filtering was applied to remove low-quality cells based on standard criteria, including retention of cells with 680–6,630 detected genes, fewer than 56,000 UMIs, and a mitochondrial transcript fraction below 20%. Ambient RNA contamination was estimated and removed using DecontX (celda v1.22.0) (18). After filtering, data were normalized by library size and log-transformed. Highly variable genes (HVGs) were selected for downstream analysis. Dimensionality reduction was performed using principal component analysis (PCA), with batch effects across 10 patient samples corrected using Harmony (v1.2.0) (19). Graph-based clustering was applied to the corrected data, and uniform manifold approximation and projection (UMAP) was used for visualization. In total, 59,869 high-quality single cells from 10 HCC patient samples were retained for analysis.

### Cell type annotation and TLS-associated immune-cell composition

Cell identities were initially assigned using SingleR (v2.0.0) (20) with the Human Primary Cell Atlas as the reference, followed by manual refinement based on canonical immune markers. Major immune lineages, including T cells (CD3E), B cells (MS4A1), and dendritic cells, were identified. Additionally, several non-immune cell populations were annotated, including hepatocytes (ALB, AFP), NK cells (NCAM1, KLRD1), monocytes (CD14, LST1), and endothelial cells (PECAM1, VWF). TLS-associated immune-cell subsets were further annotated, including regulatory T cells (FOXP3 and IL2RA), naïve and activated B cells (IGHD and SELL; CD83 and MYC), cDC1 (CLEC9A and XCR1), and cDC2 (CD1C and FCER1A). Cell populations were annotated using a combination of classical marker genes and those derived from the SingleR reference dataset.

### Module scoring and bulk deconvolution

Curated, lineage-specific, literature-derived gene sets (**Supplementary Table 2**) were used to compute single-cell TLS-promoting (PromScore) and TLS-suppressive (InhibScore) module scores using AddModuleScore in Seurat (v5.0.0). For each gene set, we evaluated gene-wise mean expression, coefficient of variation (CV), and the proportion of expressing cells (expression > 0), and retained genes with mean expression > 0.01 and expression in 5–80% of cells. Per-cell PromScore and InhibScore values were then calculated using the filtered gene sets.

Pseudo-bulk reference profiles were derived from scRNA-seq data by aggregating expression within each of the six TLS-associated immune components (TLS6). To harmonize expression levels between bulk RNA-seq and scRNA-seq, expression matrices were adjusted for a “bulk vs scRNA-seq” batch factor using removeBatchEffect (limma v3.56.2) (21). Batch-adjusted reference profiles and bulk mixtures were expressed as TPM and supplied to BayesPrism (v2.2.2) (22), which was run with MAP optimization and Gibbs sampling to obtain posterior estimates of TLS6 component fractions at the sample level.

### Construction and validation of the prognostic model

Survival associations of individual variables were first evaluated using the Kaplan–Meier method. To handle compositional constraints, an isometric log-ratio (ILR) balance score was computed as the log-ratio of the geometric mean of a focal component to the geometric mean of the remaining immune components. For stratified analyses, patients were dichotomized using the median ILR balance score as the cutoff. Univariate Cox regression was then performed to assess the association of the ILR balance score with survival. Following this, the ILR balance scores of TLS6 components were used as candidate predictors in a LASSO-penalized Cox model fitted with glmnet (v4.1.10) (23). Predictive variables with non-zero coefficients at the selected λ_min were retained, and the resulting coefficients were used to derive the TLS RiskScore as a linear combination of the selected ILR balance scores. Performance was assessed using time-dependent ROC curves at 1, 2, and 3 years computed with timeROC (v0.4) (24). The model was trained in the TCGA-LIHC cohort and validated in the independent ICGC LIRI-JP cohort, with model coefficients fixed in validation to avoid information leakage.

### Analysis of the anti–PD-1 cohort

The fixed-coefficient TLS RiskScore was applied to tumor specimens from the anti–PD-1–treated HCC cohort (GEO accession GSE202069). Treatment response was analyzed according to the responder/non-responder annotation provided in the original dataset. In the source study, treatment response was assessed according to RECIST 1.1, with complete response and partial response classified as responders and stable disease and progressive disease classified as non-responders. We therefore used this predefined binary response annotation for downstream analyses. Associations between the continuous TLS RiskScore and binary response status were evaluated in 17 patients with available response data using the Wilcoxon rank-sum test, and effect size was summarized using Cliff’s delta (Cliff’s Δ). For categorical analyses, patients were stratified into high- and low-RiskScore groups using the median TLS RiskScore as the cutoff. The association between TLS RiskScore group and binary response status was assessed using Fisher’s exact test. Odds ratios and ORR, calculated according to the original responder annotation, were reported.

### Multiplex immunofluorescence

Six-plex tyramide signal amplification (TSA)-based mIF (six protein markers plus DAPI) was performed on 4-µm FFPE sections using the Aifang TSA mIF kit (AFIHC037) according to the manufacturer’s instructions. Antigen retrieval was carried out in citrate buffer (pH 6.0) or EDTA buffer (pH 9.0), depending on the target antigen. The antibody panel included CD45 (Aifang, AF20018), CD4 (Aifang, AF20210), FOXP3 (Maxim, MAB-1004), CD11c (Aifang, AF20381), CD21 (Aifang, AF20013), and CD1c (HUABIO, HA722562), each used at 1:200. Sequential staining followed a standard HRP–TSA workflow, and nuclei were counterstained with DAPI.

To implement the TLS RiskScore at the protein level, we evaluated the local FFPE HCC cohort for archival tumor tissue suitable for six-plex TSA-based mIF staining and cell-level quantification. Cases for mIF validation were selected from this 113-case FFPE cohort using predefined clinicopathologic and technical criteria, resulting in 36 eligible cases for analysis. Eligible cases required H&E-confirmed primary HCC, no neoadjuvant systemic therapy before FFPE tissue acquisition, complete clinicopathologic and follow-up data, and sufficient viable tumor tissue in archival FFPE blocks with intact sections suitable for six-plex TSA-based mIF staining. Cases were excluded for mixed HCC–CCA histology or recurrent disease, insufficient or compromised tissue quality, strong tissue autofluorescence or other technical limitations that precluded reliable cell segmentation and phenotyping, or missing key outcome data. Full selection criteria are provided in the **Supplementary Methods**. TLS were identified on matched H&E sections by experienced pathologists, classified into three maturation stages according to predefined histologic criteria, and delineated as regions of interest (ROIs) for downstream analysis. Pathologic assessment was performed without reference to clinical outcomes or TLS RiskScore results. For cases with ambiguous TLS boundaries or discrepant maturation assignments, the sections were jointly reviewed by at least two experienced pathologists, and final classifications were determined by consensus before mIF quantification. Cell-level quantification was then performed using standardized segmentation pipelines with uniformly applied intensity thresholds to ensure measurement consistency across samples. Case-level values were summarized as unweighted means across all TLS regions.

Slides were imaged on the PhenoCycler-Fusion 2.0 platform (Akoya Biosciences) using uniform acquisition settings. Spectral unmixing and background subtraction were performed using the manufacturer’s default pipeline, without additional smoothing, nonlinear contrast enhancement, or gamma correction. An mIF-based TLS RiskScore was then computed from the densities of FOXP3^+^CD4^+^ regulatory T cells and CD11c^+^CD1c^+^ cDC2 cells within TLS ROIs, using the fixed coefficients from the transcriptomic TLS RiskScore model.

### Statistical analysis

All analyses were performed in R (v4.3.0). Single-cell data processing and clustering were carried out using Seurat (v5.0.0), Harmony (v1.2.0), SingleR (v2.0.0), and DecontX (celda v1.22.0). Bulk RNA-seq deconvolution was performed with BayesPrism (v2.2.2). Survival analyses and Cox models were performed using the survival package (v3.8.3) (25), with LASSO-Cox models fitted using glmnet (v4.1.10). Time-dependent ROC curves were computed with timeROC (v0.4), and Kaplan–Meier curves were generated using the survminer package (v0.5.1). For all stratified analyses, patients were categorized into high- and low-risk groups using the median value as the predefined cutoff. Differential expression analysis was conducted with limma (v3.56.2), and functional enrichment analyses were performed using clusterProfiler (v4.16.0). Data visualization was primarily performed using ggplot2 (v4.0.0).

Two-group comparisons were performed using two-sided Wilcoxon rank-sum tests. Multi-group comparisons were conducted using Kruskal–Wallis tests followed by Benjamini–Hochberg correction for multiple testing. Cox proportional hazards models reported hazard ratios with 95% confidence intervals, and proportional hazards assumptions were evaluated using Schoenfeld residuals. For differential expression and enrichment analyses, false discovery rate (FDR)-adjusted P values < 0.05 were considered statistically significant; otherwise, P < 0.05 was considered statistically significant.

## Results

### TLS structural features show inconsistent prognostic associations

**Figure 1:**
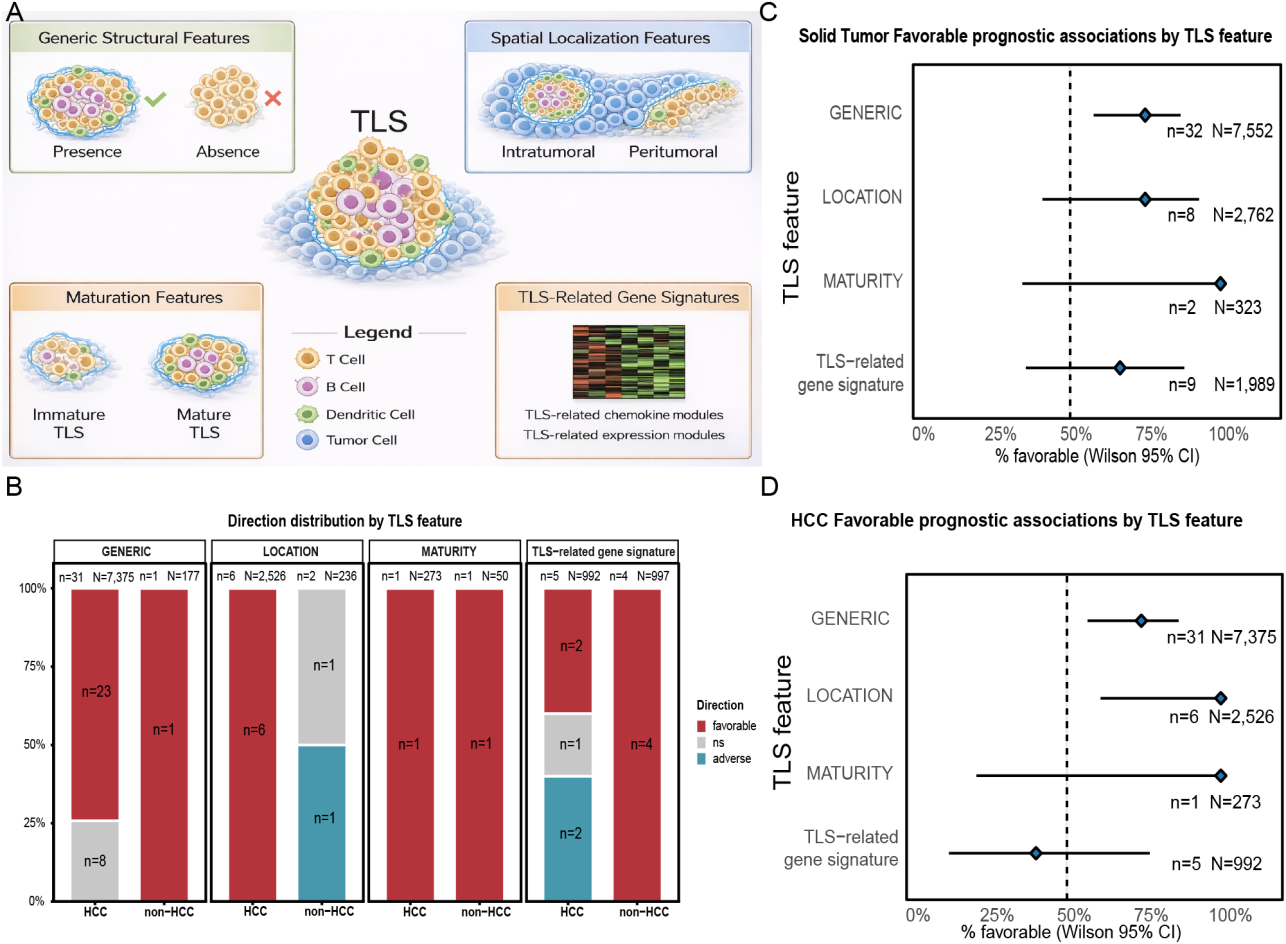
TLS structural features show inconsistent prognostic directions across studies. **(A)** Categories of TLS structural features. **(B)** Reported prognostic directions differ between HCC and non-HCC entries across categories (favorable, ns, adverse); numbers indicate entry counts (ns, not significant). **(C)** Across solid tumors, the proportion of entries reporting favorable associations varies by feature category (Wilson 95% confidence intervals); the dashed line indicates 50% favorable. n, number of study entries; N, total sample size aggregated across all entries (without adjustment for potential overlap between studies). **(D)** The HCC subset shows the same category-level summary.

Prior studies have evaluated diverse TLS structural features and their prognostic associations across solid tumors. We curated 52 TLS-prognosis entries from 16 publications; individual reports contributed multiple entries owing to distinct cohorts, centers, or TLS definitions (**Supplementary Table 1**). Reported TLS structural features were grouped into four categories: (i) generic structural features reflecting overall TLS burden (presence/absence, density, and composite scores); (ii) spatial localization (intratumoral vs peritumoral); (iii) maturation (immature vs mature); and (iv) TLS-related gene signatures derived from structurally defined TLS regions, capturing TLS-like chemokine or expression modules (**Figure 1A**).

The distribution of prognostic directions differed between HCC and non-HCC entries in a category-specific manner (**Figure 1B**). Across solid tumors, we quantified the proportion of favorable associations by category and computed Wilson 95% confidence intervals. Estimates varied widely and did not support a consistent prognostic direction (**Figure 1C**). Restricting to HCC likewise showed no consistent direction (**Figure 1D**), indicating that structural descriptors alone are insufficient for robust prognostic stratification. Structurally similar TLS may differ in immune-cell composition and associated immune programs, so we complemented structural features with TLS-associated immune-cell composition to capture variation not resolved by structural descriptors.

### Single-cell mapping of TLS-promoting and TLS-suppressive immune subsets in HCC

**Figure 2:**
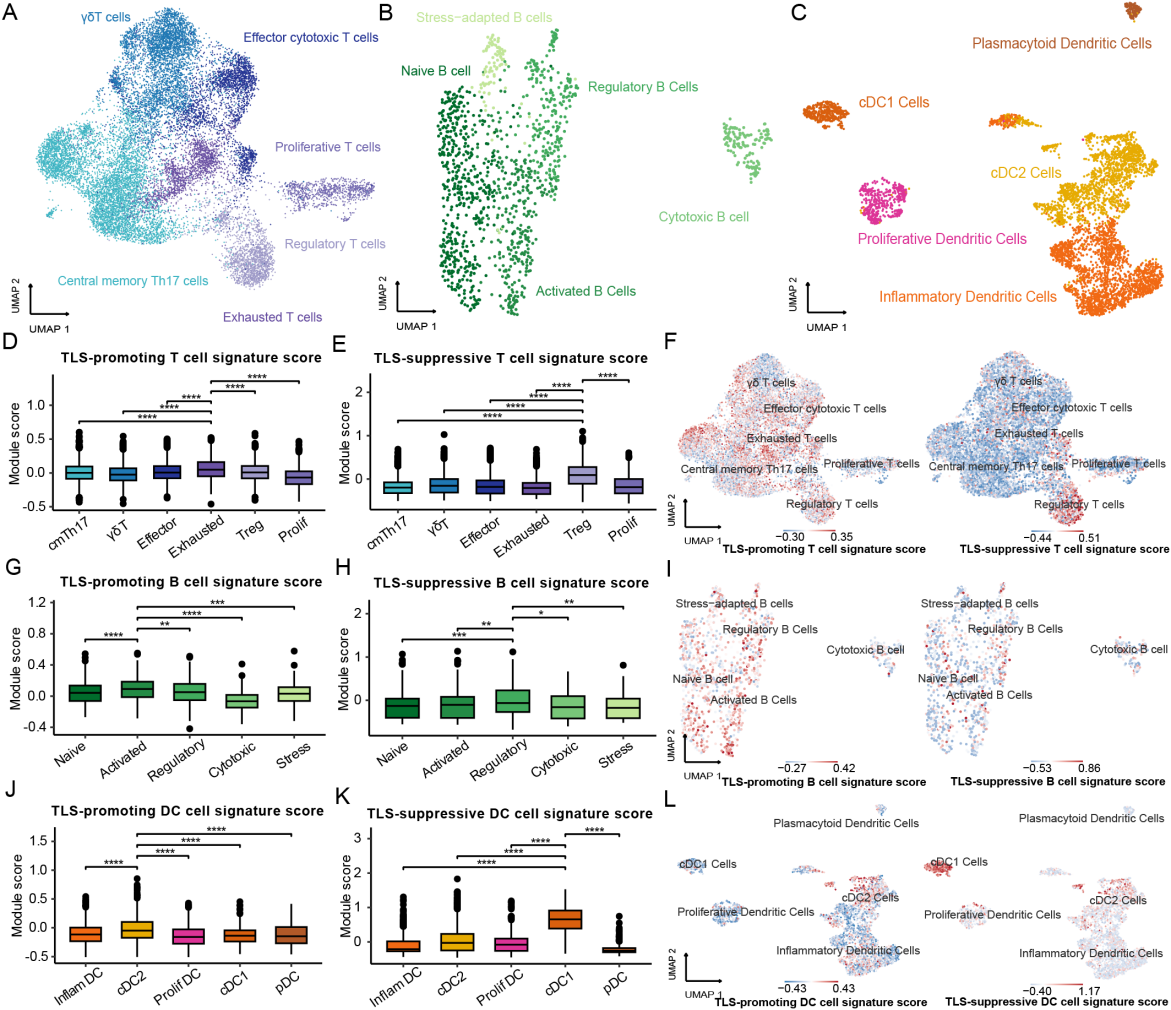
Single-cell mapping of TLS-promoting and TLS-suppressive signature scores across immune lineages in HCC. **(A–C)** UMAPs of T cells **(A)**, B cells **(B)**, and dendritic cells **(C)**, annotated by subset identity. **(D–F)** T cells: TLS-promoting **(D)** and TLS-suppressive **(E)** signature scores across subsets; UMAP colored by score **(F)**. **(G–I)** B cells: TLS-promoting **(G)** and TLS-suppressive **(H)** signature scores across subsets; UMAP colored by score **(I)**. **(J–L)** Dendritic cells: TLS-promoting **(J)** and TLS-suppressive **(K)** signature scores across subsets; UMAP colored by score **(L)**. *P < 0.05, **P < 0.01, ***P < 0.001, and ****P < 0.0001.

To profile TLS-associated immune-cell composition at cellular resolution in HCC, we analyzed a published single-cell atlas of the HCC tumor immune microenvironment (TIME) comprising 59,869 high-quality transcriptomes from 10 patients (14). Clustering identified major immune lineages and additional immune and stromal populations. Because TLS are primarily organized by T cells, B cells, and dendritic cells (6, 7, 26, 27), we focused subsequent analyses on these three lineages. T-cell subsets included central memory Th17 (CCR6, IL7R), γδ T (TRDC, TRGC1), effector cytotoxic T (NKG7, FGFBP2), exhausted T (NR4A1, TOX), regulatory T (FOXP3, IL2RA), and proliferative T (MKI67, TOP2A) cells (**Figure 2A**). B-cell subsets included naïve (IGHD, SELL), activated (CD83, MYC), cytotoxic (NKG7, PRF1), regulatory (FCRL5, DUSP4), and stress-adapted (HSPA1A, DNAJB1) B cells (**Figure 2B**). Dendritic-cell subsets included cDC1 (CLEC9A, XCR1), cDC2 (CD1C, FCER1A), proliferative dendritic cells (MKI67, TOP2A), plasmacytoid dendritic cells (CLEC4C, LILRA4), and inflammatory dendritic cells (FCN1, S100A8) (**Figure 2C**).

To relate these subsets to TLS-associated immune programs defined by literature-derived gene sets, we computed per-cell TLS-promoting and TLS-suppressive signature scores (**Supplementary Table 2**) and compared their distributions across subsets within each lineage. In T cells, both scores differed across subsets (Kruskal–Wallis; both p < 2.2 × 10^-^¹^6^): TLS-promoting scores peaked in exhausted T cells, whereas TLS-suppressive scores peaked in regulatory T cells (**Figures 2D–F**). In the present scoring framework, “TLS-promoting” denotes enrichment of literature-derived gene programs associated with TLS formation and immune-cell recruitment, rather than direct cytotoxic effector function or uniformly favorable prognostic activity. Accordingly, exhausted T cells and regulatory T cells, despite both potentially exhibiting immune-inhibitory features, were assigned to different TLS-associated categories based on their distinct enrichment of TLS-promoting versus TLS-suppressive gene programs. In B cells, scores differed across subsets (promoting, p < 2.2 × 10^-^¹^6^; suppressive, p = 0.0021), peaking in activated and regulatory B cells, respectively (**Figures 2G–I**). In dendritic cells, both scores differed across subsets (Kruskal–Wallis; both p < 2.2 × 10^-^¹^6^), with higher TLS-promoting scores in cDC2 and higher TLS-suppressive scores in cDC1 (**Figures 2J–L**). Based on these patterns, we defined six TLS-associated immune components (TLS6): three promoting components (activated B cells, exhausted T cells, and cDC2 cells) and three suppressive components (regulatory B cells, regulatory T cells, and cDC1 cells).

### Integrating TLS-associated immune-cell composition to construct a prognostic TLS RiskScore in HCC

**Figure 3:**
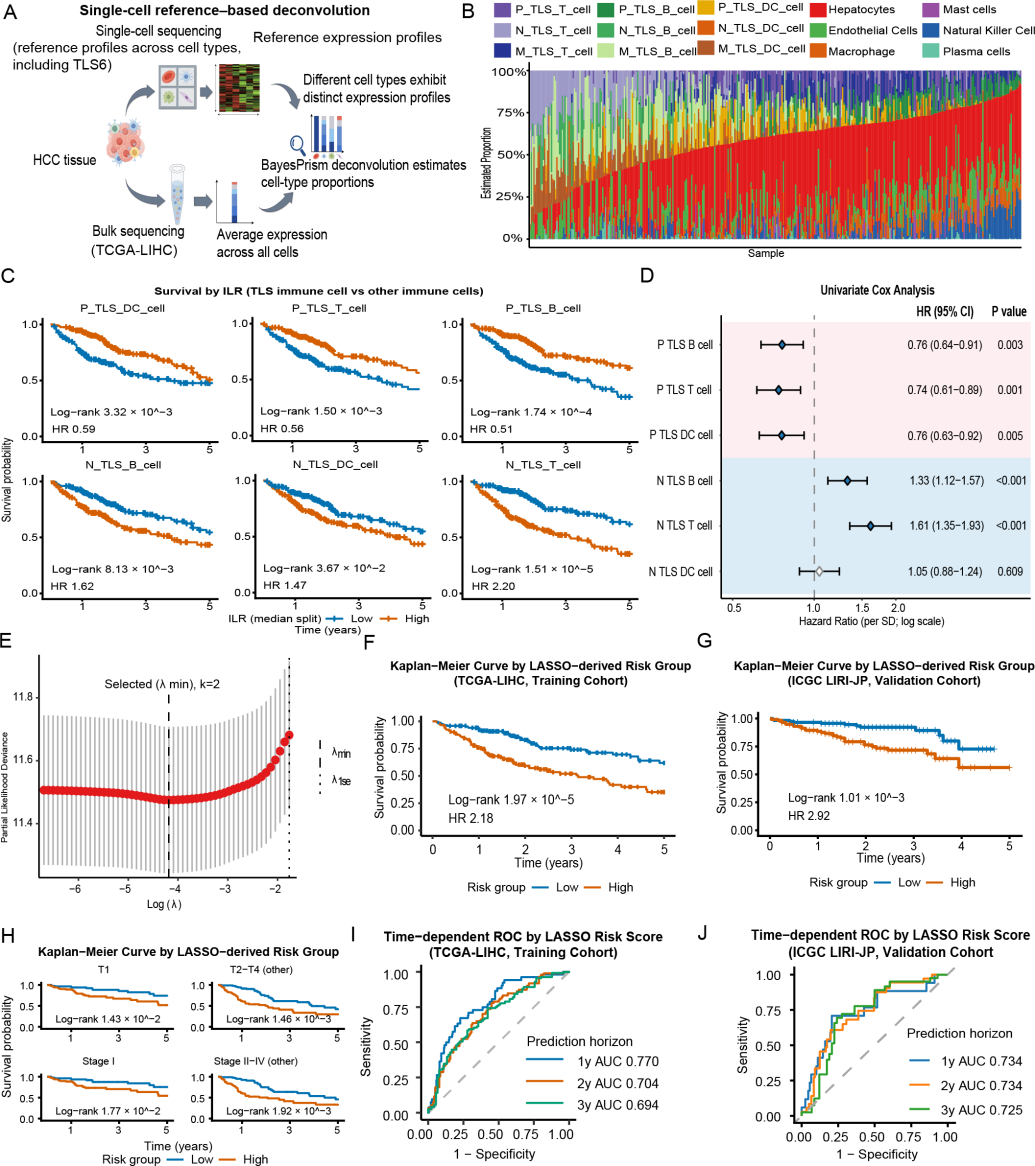
Construction of a prognostic TLS RiskScore by integrating bulk-inferred TLS-associated immune-cell composition in HCC. **(A)** Single-cell reference–based deconvolution (BayesPrism) to infer cell-type fractions from bulk RNA-seq and derive TLS6 component fractions. **(B)** Inferred fractions across TCGA-LIHC tumors (n = 342), including TLS-associated T-, B-, and dendritic-cell states and other major cell types. P_TLS, TLS-promoting components; N_TLS, TLS-suppressive components; M_TLS, lineage-specific subsets not assigned to P_TLS or N_TLS. **(C–E)** Component-level survival associations and TLS RiskScore construction: Kaplan–Meier analyses by component fraction **(C)**, univariate Cox estimates **(D)**, and LASSO–Cox feature selection **(E)**. **(F–H)** Kaplan–Meier overall survival (OS) stratified by the TLS RiskScore in TCGA-LIHC **(F)**, ICGC LIRI-JP (n = 231) **(G)**, and TCGA-LIHC pathologic strata **(H)**. **(I–J)** Time-dependent ROC curves at 1, 2, and 3 years in TCGA-LIHC **(I)** and ICGC LIRI-JP **(J)**. BayesPrism outputs are bulk-inferred cell-type fractions and do not represent *in situ* TLS composition.

We asked whether TLS6 fractions could be estimated from bulk tumor transcriptomes. Using single-cell reference expression profiles, we applied BayesPrism (22) to deconvolve TCGA-LIHC bulk RNA-seq (n = 342), estimate cell-type fractions, and extract TLS6 fractions for downstream analyses (**Figure 3A**). TLS6 fractions varied markedly across TCGA-LIHC tumors, consistent with substantial heterogeneity in inferred TLS-associated immune-cell composition (**Figure 3B**). To clarify the distribution of inferred cell fractions, we further quantified hepatocyte and total TLS6 abundances across samples. Although hepatocytes represented a major inferred cell population, they were not uniformly dominant, with a median fraction of 38.6% (interquartile range [IQR], 23.4%–49.3%) and fractions greater than 50% observed in only 24.1% of tumors. By contrast, total TLS6 fractions were measurable in most tumors, with a median of 22.1% (IQR, 14.0%–33.8%) and values greater than 10% observed in 87.1% of tumors (**Supplementary Figure 1B**), supporting the feasibility of downstream TLS6-based analyses.

We tested associations between these inferred fractions and overall survival (OS) in TCGA-LIHC. In Kaplan–Meier analyses (median split), higher TLS-promoting component fractions were associated with improved survival, whereas higher TLS-suppressive component fractions were associated w乘风陈ith worse survival (**Figure 3C**). In univariate Cox analyses, associations were significant for activated B cells (p = 0.003), exhausted T cells (p = 0.001), cDC2 cells (p = 0.005), regulatory B cells (p < 0.001), and regulatory T cells (p < 0.001), but not for cDC1 cells (p = 0.609) (**Figure 3D**). Thus, the inferred TLS-associated components showed prognostic signals in opposing directions, suggesting that an integrated assessment may better capture clinically relevant variation in TLS-associated immune-cell composition.

We fitted LASSO–Cox models using the ILR balance scores of the six TLS6 components and selected λ_min, whereas λ_1se yielded a null model (**Figure 3E**). The final model retained two components: regulatory T cells and cDC2 cells. This yielded a two-feature TLS RiskScore, with higher values indicating a predominance of Treg-weighted suppressive over cDC2-weighted promoting TLS-associated immune composition.

In TCGA-LIHC, the TLS RiskScore stratified OS (log-rank p = 1.97 × 10^-5^; HR = 2.18) (**Figure 3F**). We assessed the robustness of the TLS RiskScore across available pathologic strata in TCGA-LIHC. Stratification remained evident across TNM T categories (T1 vs T2–T4) and stage strata (I vs II–IV) (**Figure 3H**). Furthermore, we performed multivariable Cox regression analysis to evaluate whether the TLS RiskScore provides prognostic information independent of routine clinical parameters. The TLS RiskScore remained significantly associated with OS (HR = 2.657, 95% CI 1.726–4.089, P < 0.001), independent of tumor stage (HR = 1.767, 95% CI 1.171–2.664, P = 0.007) (**Supplementary Figure 1C**). Time-dependent ROC AUCs were 0.770, 0.704, and 0.694 at 1, 2, and 3 years, respectively (**Figure 3I**). We further compared the TLS RiskScore with a full six-feature Cox model constructed from all TLS6 ILR balance scores in TCGA-LIHC. The full model showed only modest, non-significant AUC improvements at 1, 2, and 3 years (ΔAUC = 0.005, 0.023, and 0.037; paired bootstrap P = 0.764, 0.222, and 0.058), with similar C-index values between the two-feature and six-feature models (0.698 vs. 0.710). In ICGC LIRI-JP (n = 231), the TLS RiskScore likewise stratified OS (log-rank p = 1.01 × 10^-^³; HR = 2.92) (**Figure 3G**) and achieved time-dependent ROC AUCs of 0.734, 0.734, and 0.725 at 1, 2, and 3 years, respectively (**Figure 3J**). Collectively, the TLS RiskScore provided robust and independent prognostic stratification in HCC and captured bulk-inferred variation in TLS-associated immune composition, suggesting accompanying differences in the inferred TLS-associated immune programs within the TIME.

### TLS RiskScore–associated immune programs across strata in HCC

**Figure 4:**
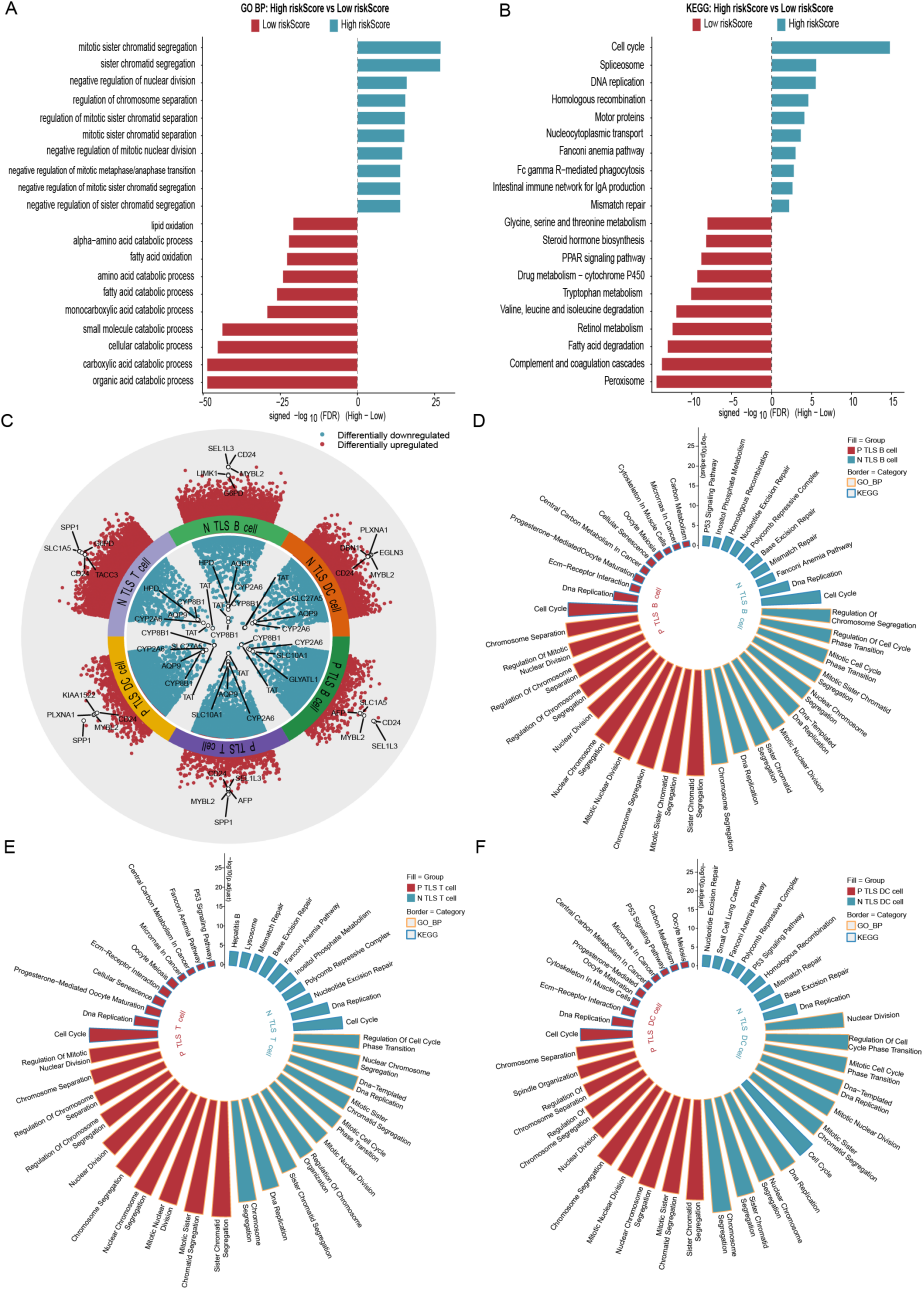
TLS RiskScore–associated immune programs across strata in HCC. **(A, B)** Pathway enrichment analyses comparing bulk tumor gene expression between low- and high-RiskScore tumors in TCGA-LIHC **(A)** GO; **(B)** KEGG. **(C)** BayesPrism-inferred cell-type–resolved expression contributions used for compartment-specific differential expression in B-cell, T-cell, and dendritic-cell compartments. **(D–F)** Compartment-specific pathway enrichment analyses based on cell-type–resolved differential expression **(D)** B-cell; **(E)** T-cell; **(F)** dendritic-cell.

To assess whether TLS RiskScore strata capture distinct immune programs within the TIME, we compared bulk tumor gene expression between low- and high-RiskScore TCGA-LIHC tumors and summarized pathway-level differences (**Figures 4A, B**).

GO and KEGG enrichment analyses in TCGA-LIHC revealed contrasts between TLS RiskScore strata (**Figures 4A, B**). High-RiskScore tumors were enriched for mitotic and chromosome-segregation processes and for DNA replication and repair pathways (cell cycle, DNA replication, and homologous recombination), whereas low-RiskScore tumors were enriched for oxidative and catabolic metabolic processes (peroxisome and fatty acid degradation) and complement and coagulation cascades (**Figures 4A, B**).

Because bulk enrichment is not cell-type-specific, we used cell-type–resolved expression contributions estimated by BayesPrism—derived from the same deconvolution used to infer TLS6 fractions—to assess RiskScore-associated differential expression within B-cell, T-cell, and dendritic-cell compartments (**Figure 4C**). We then performed compartment-specific GO and KEGG enrichment analyses (**Figures 4D–F**). Although enrichment was assessed in both strata, we highlight pathways enriched in high-RiskScore tumors to facilitate interpretation of immune programs associated with poor prognosis. In high-RiskScore tumors, TLS-promoting components across B-cell, T-cell, and dendritic-cell compartments were enriched for proliferation-related programs, including DNA replication and mitotic chromosome segregation in B cells (**Figure 4D**), mitotic cell-cycle programs in T cells (**Figure 4E**), and cell-cycle and mitotic programs in dendritic cells (**Figure 4F**), indicating a shared proliferation-associated signature. In contrast, TLS-suppressive components were enriched for genome-stability and checkpoint-associated programs. In B cells and dendritic cells, enrichment involved p53 signaling together with DNA damage–response and repair pathways (**Figures 4D, F**). In T cells, enrichment involved DNA repair pathways (base excision repair, mismatch repair, and nucleotide excision repair) and regulation of cell-cycle phase transitions (**Figure 4E**). These patterns suggest shared enrichment of DNA damage–response and cell-cycle control programs, consistent with increased genome maintenance across TLS-suppressive components. Together, TLS RiskScore strata captured differences in TLS-associated composition and immune programs associated with TLS6 components within the TIME.

### TLS RiskScore is associated with response to PD-1 blockade in HCC

**Figure 5:**
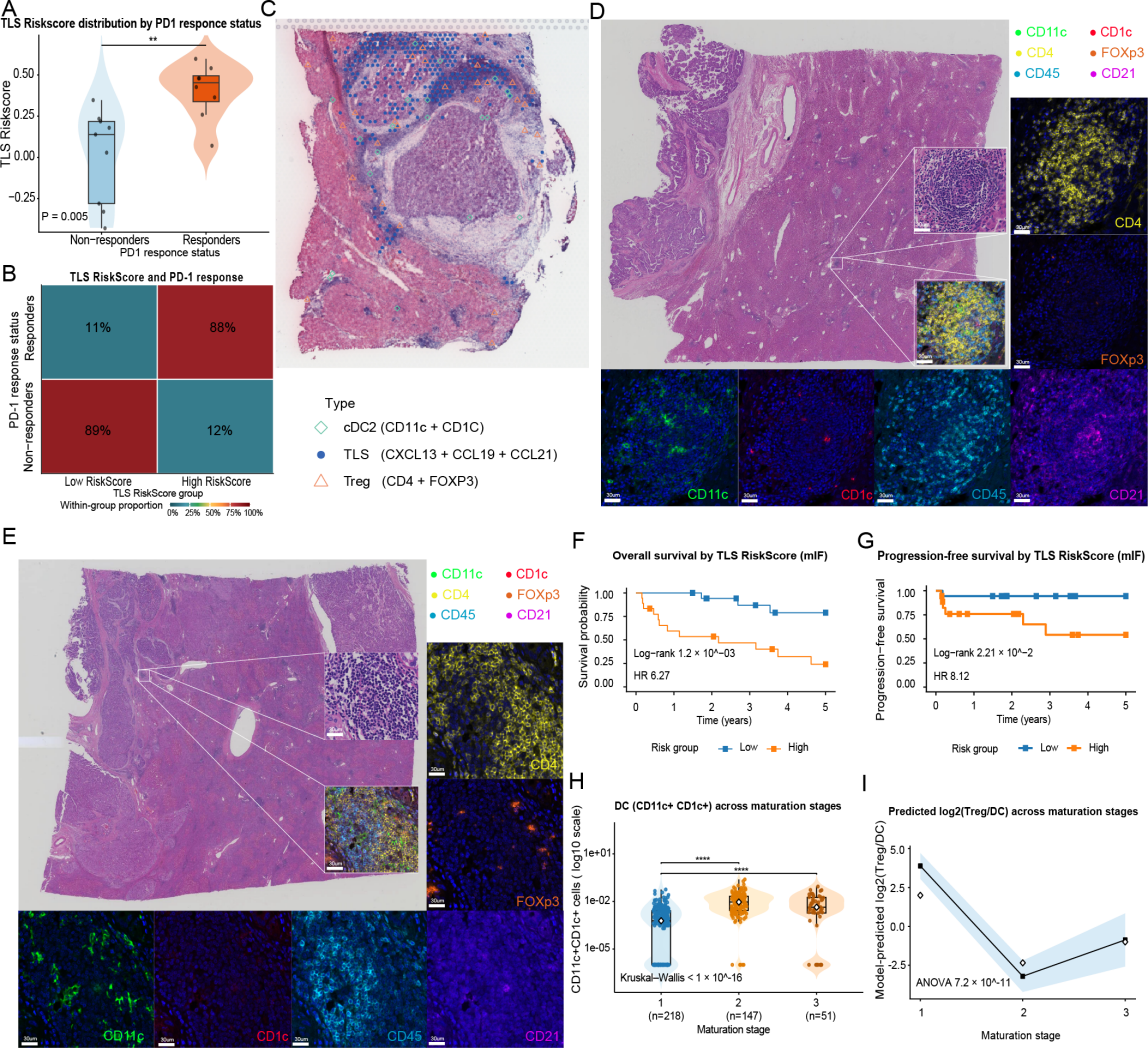
TLS RiskScore association with response to PD-1 blockade and protein-level implementation in FFPE HCC. **(A, B)** External anti–PD-1-treated HCC cohort: TLS RiskScore by response status **(A)** and response rates by TLS RiskScore group using the median cutoff **(B)**. In **(B)**, colors indicate the within-group proportions of responders and non-responders within each TLS RiskScore group. **(C)** Spatial transcriptomics mapping canonical TLS-associated chemokines (CXCL13, CCL19, CCL21) and signature signals for regulatory T cells (Treg) and conventional type 2 dendritic cells (cDC2) in representative HCC sections. **(D, E)** Representative TLS regions in FFPE HCC illustrating cDC2-predominant versus Treg-predominant cellular composition by H&E and multiplex immunofluorescence (mIF) staining (CD11c, CD1c, CD4, FOXP3, CD45, CD21). **(F, G)** Overall survival (OS) **(F)** and progression-free survival (PFS) **(G)** stratified by an mIF-based TLS RiskScore derived from TLS-region Treg (CD4^+^FOXP3^+^) and cDC2 (CD11c^+^CD1c^+^) measurements (median cutoff). **(H)** cDC2 fractions across TLS maturation stages. **(I)** Treg-to-cDC2 ratio across TLS maturation stages. **P < 0.01 and ****P < 0.0001.

Previous studies have reported associations between TLS-related gene signatures and clinical benefit from immune checkpoint blockade (28–30), including PD-1 blockade (12, 31), suggesting that the TLS RiskScore may be relevant to immunotherapy response. To assess whether the TLS RiskScore is associated with response to PD-1 blockade, we analyzed tumor specimens from an external anti–PD-1–treated HCC cohort (n = 17) and calculated scores using the prespecified formula. Responders had higher TLS RiskScores than non-responders (Wilcoxon P = 0.005; Cliff’s Δ = 0.83) (**Figure 5A**). Using the median TLS RiskScore as the cutoff, the overall response rate (ORR) was 88% in the high-RiskScore group versus 11% in the low-RiskScore group (odds ratio, 35.89; Fisher’s exact P = 0.003) (**Figure 5B**). These preliminary analyses suggest that higher TLS RiskScores may be associated with a higher response rate to PD-1 blockade. Because the score was originally developed for survival-based risk stratification, this treatment-response association should be interpreted as exploratory. This observation further motivated our subsequent assessment of the spatial localization of TLS-localized Treg and cDC2 cells in routine FFPE HCC specimens.

### Protein-level implementation of the TLS RiskScore in FFPE HCC

To assess feasibility of implementing the TLS RiskScore in routine FFPE HCC tissue, we performed spatial transcriptomic analysis on representative HCC sections. Hotspots of canonical TLS-associated chemokines (CXCL13, CCL19, and CCL21) overlapped with regions enriched for Treg (CD4^+^FOXP3^+^) and cDC2 (CD11c^+^CD1c^+^) signatures (**Figure 5C**). These overlaps support localizing the RiskScore-defining Treg and cDC2 components to chemokine hotspots and motivate protein-level quantification within histology-defined TLS regions of interest in FFPE tissue.

Accordingly, we performed multiplex immunofluorescence (mIF) on FFPE samples from 36 eligible resected HCC cases selected from the local multicenter FFPE cohort according to predefined clinicopathologic and technical criteria. TLS regions of interest were identified on matched H&E sections and registered to mIF images. Within these regions, we quantified the proportions of CD4^+^FOXP3^+^ regulatory T cells and CD11c^+^CD1c^+^ cDC2 cells using standardized cell segmentation and uniformly applied intensity thresholds (**Figures 5D, E**). We then calculated an mIF-based TLS RiskScore from these TLS-region measurements using coefficients predefined by the transcriptomic model, without refitting. Using the median mIF-based TLS RiskScore as the cutoff, the high-RiskScore group had worse OS (log-rank P = 1.2 × 10^-^³; HR ≈ 6.3) and worse progression-free survival (PFS) (log-rank P = 2.2 × 10^-^²; HR ≈ 8.1) than the low-RiskScore group (**Figures 5F, G**).

Across 36 cases, we identified 416 TLS and classified them into maturation stages (9): stage 1 (lymphoid aggregates), stage 2 (follicle-like TLS without germinal center features), and stage 3 (follicle-like TLS with germinal center features). With maturation, TLS cDC2 fractions increased markedly (**Figure 5H**), whereas Treg fractions changed more modestly (**Supplementary Figure 1D**). Consistently, the modeled Treg-to-cDC2 ratio was highest in stage 1, lowest in stage 2, and intermediate in stage 3 (**Figure 5I**). Together, these results support a proof-of-concept protein-level implementation of the TLS RiskScore in FFPE HCC tissue and show that TLS maturation is associated with stage-dependent differences in the Treg and cDC2 components underlying the TLS RiskScore.

## Discussion

Across cancers, TLS have been widely evaluated through structural features associated with antitumor immunity; however, clinical associations remain inconsistent when TLS are assessed primarily by these features alone. In our systematic review of studies evaluating TLS in solid tumors, commonly used TLS structural features and TLS-related gene signatures—including presence, density, location, and maturation—did not show a consistent prognostic direction across cohorts. This inconsistency is also evident in HCC, where prior studies have reported divergent associations between TLS features and clinical outcomes (2, 9, 30). These observations suggest that TLS structural features alone are insufficient to capture the immune-cell composition and immune programs that characterize broader TIME states. The TLS RiskScore developed here therefore differs from previous HCC TLS assessments by focusing on the relative balance of TLS-associated immune components and linked immune programs, rather than on TLS structural features or bulk TLS-related gene expression alone. Thus, the TLS RiskScore is intended to complement existing TLS scoring approaches as a TIME-state summary based on TLS-associated immune-cell composition and programs.

To address this limitation in HCC, we translated this interpretive shift into a computable model based on TLS-associated immune components and programs. We operationalized this concept by defining six TLS-associated immune components (TLS6) and applying the TLS6 reference in BayesPrism deconvolution of TCGA-LIHC bulk RNA-seq to infer their relative abundances. Across tumors, inferred TLS6 profiles varied substantially: TLS-promoting components tended to be associated with better survival, whereas TLS-suppressive components tended to be associated with worse survival. We compressed these associations into a two-feature TLS RiskScore, with components selected by LASSO-Cox, dominated by a Treg-weighted suppressive component and a cDC2-weighted promoting component, consistent with prior evidence that immunoregulatory T-cell infiltration can attenuate TLS-associated antitumor immunity (10) and that cDC2-linked helper programs support GC-like TLS organization (27, 30). In TCGA-LIHC, higher TLS RiskScores were associated with poorer overall survival. Importantly, this compression captured differences not only in inferred TLS-associated immune-cell composition but also in immune programs attributable to TLS6 components across B-cell, T-cell, and dendritic-cell compartments. TLS-promoting components were preferentially enriched for proliferation-associated programs, whereas TLS-suppressive components were enriched for genome-integrity and cell-cycle checkpoint programs. In an independent anti–PD-1-treated HCC cohort, the TLS RiskScore also showed a preliminary association with clinical response to PD-1 blockade. Together, these findings support the premise that interpreting TLS through TLS-associated immune components and programs provides a useful framework for understanding clinically relevant TIME states in HCC.

The apparently divergent prognostic and treatment-response associations of the TLS RiskScore should be interpreted in relation to treatment setting. Because the score was developed using survival-based Cox modeling, it represents a prognosis-oriented risk score rather than a direct measure of immunotherapy resistance. Thus, a high TLS RiskScore indicates poorer prognosis in survival cohorts but does not necessarily imply resistance to PD-1 blockade. One possible interpretation is that a Treg-enriched TLS-associated state reflects an immune-active but restrained microenvironment, in which pre-existing antitumor immune activity coexists with compensatory immune regulation. This interpretation is supported by prior evidence that TLS-related chemokine programs can mark robust immunogenic activity and pre-existing immunosurveillance (11), while TLS function is shaped by the balance of immune-activating and immunoregulatory cellular components (30, 32). Under natural disease conditions, such restrained effector immunity may be insufficient for durable tumor control, whereas PD-1 blockade may partially release pre-existing checkpoint-regulated immune activity. This possibility is consistent with studies linking TLS-positive or immune-infiltrated tumors to improved immune checkpoint blockade response (6, 10). Therefore, the association between higher TLS RiskScores and PD-1 response should be viewed as a preliminary observation that may depend on the treatment setting, rather than evidence that the score directly measures immunotherapy sensitivity.

To translate this transcriptome-derived TIME-state summary into routinely assessable tissue measurements, we implemented a protein-based TLS RiskScore in FFPE HCC samples collected across multiple centers. Using multiplex immunofluorescence, we quantified TLS-localized CD4^+^FOXP3^+^ regulatory T cells and CD11c^+^CD1c^+^ cDC2 cells. By applying the fixed transcriptomic coefficients to these protein-based fractions without refitting, we showed that the TIME-state summary captured by the TLS RiskScore is not restricted to transcriptomic measurements but can be reproduced at the protein level, yielding similar stratification of OS and PFS. To relate the protein-based TLS RiskScore to conventional structural assessment, we examined TLS maturation in relation to TLS-localized Treg and cDC2 composition. Across 36 cases (416 TLS analyzed), maturation was accompanied by a marked increase in the cDC2 fraction, whereas the Treg fraction changed more modestly, producing a stage-dependent pattern in the Treg-to-cDC2 ratio. These maturation-linked shifts provide composition-level context for interpreting maturation-based structural assessment in relation to the TLS RiskScore.

By explicitly modeling TLS-associated immune-cell composition and immune programs, our study provides a methodological framework that helps to contextualize divergent prognostic and therapeutic associations of TLS reported in HCC. It positions TLS evaluation as a TIME-state summary that integrates TLS-associated immune-cell composition with associated immune programs, complementing conventional TLS structural assessment. Practically, it supports a compact, interpretable score that can be derived from bulk transcriptomes and implemented in FFPE tissue without refitting, enabling reproducible evaluation across datasets and tissue workflows. Nonetheless, several limitations warrant consideration. First, our analyses relied primarily on reference-based deconvolution of bulk transcriptomes; therefore, the TLS-associated immune components should be interpreted as inferred transcriptomic estimates rather than direct *in situ* measurements of spatial architecture. Second, the maturation-associated patterns observed via mIF remain descriptive and require validation in larger spatial cohorts. Most importantly, the immunotherapy-response analysis was limited by the small size of the anti–PD-1-treated cohort (n = 17) and the instability of odds ratio estimates based on few response events. Although response status in the source study was assigned according to RECIST 1.1, our analysis relied on the predefined responder/non-responder annotation rather than independent radiologic reassessment. Therefore, the association between the TLS RiskScore and response to PD-1 blockade should be considered a preliminary and exploratory finding rather than definitive evidence of predictive value. Future work should prioritize prospective validation in larger immunotherapy-treated cohorts, standardized TLS segmentation, and larger-scale spatial or *in situ* profiling to strengthen the link between this TIME-state summary and spatially resolved TLS biology in HCC.

## Data Availability

The datasets presented in this study can be found in online repositories. The names of the repository/repositories and accession number(s) can be found in the article/**Supplementary Material**.

## Glossary

cDC2: conventional type 2 dendritic cells
CI: confidence interval
FFPE: formalin-fixed paraffin-embedded
HCC: hepatocellular carcinoma
HR: hazard ratio
ILR: isometric log-ratio
mIF: multiplex immunofluorescence
ORR: overall response rate
OS: overall survival
PFS: progression-free survival
ROI: region of interest
TIME: tumor immune microenvironment
TLS: tertiary lymphoid structures
Treg: regulatory T cell
