# Supplementary material for: Tumor immune microenvironment states inferred from TLS-associated immune-cell composition stratify prognosis in hepatocellular carcinoma
Source: Front Immunol. 2026 May 28;17:1818138. doi: 10.3389/fimmu.2026.1818138 (PMC13253696; doi:10.3389/fimmu.2026.1818138)
Supplement: Supplementary file 1 [file DataSheet1.docx]

**Tumor Immune Microenvironment States Inferred from TLS-Associated Immune-Cell Composition Stratify Prognosis in Hepatocellular Carcinoma**

Chihyuan Cheng^1,3,4^, Geng Chen^2,4^, Jing Zhang^3,4^, Liman Qiu^2^, Zhenli Li^2^, Xiuqing Dong^2^, Chenfan Lu^2^, Qiming Wu^2^, Xiaohui Peng^2^, Zhixiong Cai^2^*and Yongyi Zeng^1,2^*

**Author details**

^1^ The First Affiliated Hospital of Fujian Medical University, Fuzhou, China

^2^ The United Innovation of Mengchao Hepatobiliary Technology Key Laboratory of Fujian Province & Mengchao Hepatobiliary Hospital of Fujian Medical University, Fuzhou, China

^3^ The Fifth Hospital of Xiamen, Xiamen, China.

^4^ These authors contributed equally to this work.

*** Correspondence Authors:**

**Prof. Zhi-Xiong Cai,** PhD, The United Innovation of Mengchao Hepatobiliary Technology Key Laboratory of Fujian Province, Mengchao Hepatobiliary Hospital of Fujian Medical University, No.312, Xihong Road, Fuzhou, Fujian Province, 350025, China. E-mail: caizhixiong1985@163.com; ORCID: 0000-0002-0912-8372.

**Prof. Yong-Yi Zeng,** MD, The First Afﬁliated Hospital of Fujian Medical University, Fuzhou, China, 20 Chazhong Road, Fuzhou, Fujian Province, 350005 China. The United Innovation of Mengchao Hepatobiliary Technology Key Laboratory of Fujian Province, Mengchao Hepatobiliary Hospital of Fujian Medical University, No.312, Xihong Road, Fuzhou, Fujian Province, 350025, China. E-mail: [lamp197311@126.com](mailto:lamp197311@126.com)

Supplementary material

**Supplementary methods**

*Single-Cell Data Processing*: Raw UMI count matrices from 10x Genomics scRNA-seq (GEO: GSE149614) were processed using Seurat (v5.0.0). Ambient RNA contamination was estimated using DecontX (celda v1.22.0) applied to raw UMI counts, and cells with contamination scores in the top decile were excluded. Cells were then filtered to retain 680–6,630 detected genes, <56,000 UMIs, and <20% mitochondrial transcripts. Doublet removal was not performed because downstream analyses focused on major immune lineages and TLS-associated subsets rather than fine-grained subclustering. Filtered data were log-normalized (scale factor 10,000), variable genes were selected (vst; 2,000 features), and PCA was performed. Harmony integration was applied to the PCA embeddings (top 26 components) to mitigate inter-patient batch effects, followed by graph-based clustering (resolution 0.8) and UMAP visualization using the Harmony embeddings (dims 1–26).

*Cell Type Annotation*: Initial cell-type annotation was performed using SingleR (v2.0.0) with the Human Primary Cell Atlas reference provided by the celldex package. SingleR was applied in a cluster-based manner using Seurat-derived cluster identities, and reference labels were used for initial annotation. Automated annotations served as a preliminary guide and were subsequently refined through marker-based review using canonical lineage markers to resolve closely related immune populations and generate the final cell-type labels used in downstream analyses.

*TLS module scoring and definition of TLS-associated immune components (TLS6)*: This section provides extended technical details supporting the TLS6 definition described in the main text. Lineage-specific TLS-promoting and TLS-suppressive gene sets were curated from the literature (Supplementary Table 2) and used to compute per-cell module scores in the scRNA-seq data. Gene symbols were matched to scRNA-seq feature names; duplicated entries were collapsed to unique gene symbols, and genes absent from the expression matrix were excluded.

To minimize the influence of rarely detected or ubiquitously expressed genes, gene sets were filtered using prespecified criteria based on Seurat log-normalized expression values (RNA assay, the Seurat data layer). Genes were retained if they exhibited a mean expression >0.01 and were detected in 5%–80% of cells (expression >0). These filtering criteria were applied uniformly across all TLS-promoting and TLS-suppressive gene sets.

Per-cell module scores were calculated using Seurat AddModuleScore, with filtered gene sets supplied as feature lists and default settings used for control gene selection. Module score distributions were compared across subsets defined by the primary clustering and annotation within each major lineage (T cells, B cells, and dendritic cells). Statistical differences were assessed using Kruskal–Wallis tests, followed by within-lineage interpretation of subsets exhibiting dominant TLS-promoting or TLS-suppressive score patterns. Based on these patterns, subsets representing TLS-promoting and TLS-suppressive programs were selected from each lineage and defined as the six TLS-associated immune components (TLS6)—activated B cells, regulatory B cells, exhausted T cells, regulatory T cells, cDC1, and cDC2—used for downstream deconvolution and model construction.

*Pseudobulk reference construction and harmonization for deconvolution*: For bulk deconvolution, TCGA-LIHC bulk RNA-seq profiles were restricted to tumor samples by excluding non-tumor (Normal) specimens based on clinical annotations. To mitigate platform-related differences between bulk RNA-seq and scRNA-seq data prior to deconvolution, bulk and single-cell expression matrices were harmonized. Specifically, bulk and scRNA-seq expression values were normalized using the same log-normalization scheme and restricted to the intersecting gene set used for downstream deconvolution.

Batch effects attributable to data type (bulk vs scRNA-seq) were adjusted using the removeBatchEffect function in the limma package with a two-level batch indicator applied to the merged log-normalized expression matrix. The batch-corrected values were subsequently transformed back to linear scale, and negative values introduced by batch correction were truncated to zero.

Pseudobulk reference profiles were constructed from the scRNA-seq data by averaging expression within each TLS-associated immune component (TLS6). The batch-corrected bulk expression matrix was used as the mixture input for downstream BayesPrism deconvolution.

*BayesPrism deconvolution and downstream outputs:* BayesPrism (v2.2.2) was applied to infer TLS6 component fractions in bulk TCGA-LIHC tumor transcriptomes using scRNA-seq–derived reference profiles. Deconvolution was performed at the level of the six TLS-associated immune components (TLS6), which were treated as the reference cell types.

Pseudobulk reference profiles were generated by averaging scRNA-seq gene expression across cells assigned to each TLS6 component, and TCGA-LIHC bulk tumor expression profiles were used as the mixture input. Reference and mixture matrices were provided to BayesPrism on a TPM-scale (non–log-transformed) expression basis, after restriction to the intersecting gene set to ensure a consistent feature space.

BayesPrism was run using Gibbs sampling with parallel computation. Two categories of outputs were used for downstream analyses. First, posterior mean TLS6 component fractions (type-level estimates) were extracted and used for survival association analyses and construction of the TLS RiskScore. Second, BayesPrism-derived TLS6 component–resolved expression contributions were used to support compartment-specific differential expression analyses by focusing on expression attributable to B-cell, T-cell, and dendritic-cell compartments, followed by pathway enrichment analyses as described below.

*Survival association testing and TLS RiskScore construction*: Overall survival (OS) analyses were conducted in TCGA-LIHC tumor samples with available clinical follow-up and deconvolution outputs. Survival time was defined as days to death when available and otherwise as days to last follow-up, and was converted to years for analysis. Survival status was coded as 1 for death and 0 for censored observations (alive at last follow-up). Samples with follow-up time ≤30 days were excluded.

To assess OS associations of individual TLS-associated immune components under compositional constraints, isometric log-ratio (ILR) balances were computed. For each focal TLS component, the ILR balance was defined as the natural logarithm of the ratio between the focal component and the geometric mean of the remaining immune components included in the analysis. A small pseudocount (1 × 10⁻⁶) was added to component fractions prior to geometric-mean and log-ratio calculations to avoid undefined values due to zeros. Univariate Cox proportional hazards models were fitted for each ILR balance to evaluate associations with OS.

For multivariable feature selection and score construction, a LASSO-penalized Cox proportional hazards model (alpha = 1) was fitted using glmnet with cross-validation, and the penalty parameter was selected at λ_min. The TLS RiskScore was defined as the linear predictor (RiskScore = Σ β_i × ILR_i) using the non-zero coefficients from the selected model. For Kaplan–Meier analyses, patients were stratified into high- and low-RiskScore groups using the median RiskScore as the cutoff. Performance was assessed using time-dependent ROC analyses at 1, 2, and 3 years.

*Differential expression and enrichment analyses*: Differential expression analyses were performed to characterize transcriptional differences between TLS RiskScore strata in TCGA-LIHC tumors, using a median RiskScore cutoff. For bulk-level analyses, gene expression profiles were compared between high- and low-RiskScore tumors, and differentially expressed genes were identified to support pathway-level interpretation.

To localize RiskScore-associated transcriptional programs to major immune compartments, BayesPrism-derived cell-type–resolved expression contributions (bulk-inferred, cell-type–attributed signals) were further analyzed. Expression attributable to the B-cell, T-cell, and dendritic-cell compartments was compared between high- and low-RiskScore tumors, and compartment-specific differentially expressed genes were identified.

For both bulk and compartment-specific analyses, differential expression was performed using a linear modeling framework with empirical Bayes moderation and multiple-testing correction. Functional interpretation was conducted using Gene Ontology biological process and KEGG pathway enrichment analyses on genes upregulated in each RiskScore stratum, and enriched terms were summarized for visualization and interpretation.

*Anti–PD-1–treated HCC cohort analysis (GSE202069)*: To evaluate the association between the TLS RiskScore and response to PD-1 blockade, an independent anti–PD-1–treated HCC cohort (GEO: GSE202069) was analyzed. As described in the original study, samples were retrospectively collected from the biobank of Nanjing Drum Tower Hospital between January 2019 and June 2021. Seventeen patients with available clinical response data to anti–PD-1 therapy and pretreatment tumor specimens obtained by biopsy or surgical resection were included (8 responders and 9 non-responders).

For each pretreatment tumor, the TLS RiskScore was calculated using the prespecified model structure and fixed coefficients derived from the training cohort, without coefficient updating or model refitting. Associations between the continuous TLS RiskScore and treatment response (responder vs non-responder) were assessed using a two-sided Wilcoxon rank-sum test, and effect size was summarized using Cliff’s delta (Cliff’s Δ). For categorical analyses, patients were stratified into high- and low-RiskScore groups using the within-cohort median RiskScore as the cutoff. Associations between RiskScore group and response status were evaluated using Fisher’s exact test, with odds ratios and 95% confidence intervals reported alongside group-wise response rates.

*Spatial transcriptomics analysis and TLS-core localization (Fig. 5C)*: Processed spatial transcriptomics (ST) data were analyzed in Seurat. Within each tissue section, TLS-seed spots were defined by concurrent expression of canonical TLS chemokines (CXCL13, CCL19, and CCL21; expression >0). To visualize immune proxy signals, Treg-proxy spots were defined by joint expression of CD4 and FOXP3, and cDC2-proxy spots by joint expression of ITGAX and CD1C. For each proxy marker, the positivity threshold was set to the median expression among non-zero spots within that section to reduce sensitivity to sparsity.

To identify dense TLS regions (“TLS core”), local neighborhood density was computed among TLS-seed spots. A neighborhood radius was defined adaptively for each section as a fixed multiple of the median nearest-neighbor distance between TLS-seed spots, and TLS-core spots were defined as TLS-seed spots exceeding a prespecified local density threshold within this radius.

For Fig. 5C, hematoxylin and eosin (H&E) images were overlaid with the four spot categories (TLS seeds, TLS core, Treg-proxy, and cDC2-proxy) to visualize their spatial co-localization in representative HCC sections.

*Definition of the mIF cohort and selection criteria for Multiplex immunofluorescence (mIF)*: The two-stage screening process for the multicenter FFPE cohort is described in the main text. The following prespecified inclusion and exclusion criteria were used to define the multiplex immunofluorescence (mIF) cohort.

Inclusion criteria were: (i) primary hepatocellular carcinoma (HCC) confirmed by hematoxylin and eosin (H&E) review; (ii) no neoadjuvant systemic therapy before FFPE tissue acquisition; (iii) complete clinicopathologic and follow-up data, including baseline characteristics, treatments, and outcome endpoints; and (iv) sufficient viable tumor tissue in archival FFPE blocks, with intact sections that met the technical requirements of the six-marker mIF panel.

Exclusion criteria were: (i) mixed hepatocellular carcinoma–cholangiocarcinoma (HCC–CCA) histology or recurrent disease; (ii) insufficient tissue or compromised section integrity due to extensive necrosis or hemorrhage, decalcification, or suboptimal fixation; (iii) strong tissue autofluorescence or other technical limitations that precluded reliable cell segmentation and phenotyping; and (iv) missing key outcome data. Application of these criteria to the screened FFPE cohort (n = 113) resulted in 36 eligible cases for mIF analysis.

*Multiplex immunofluorescence and image quantification*: Multiplex immunofluorescence (mIF) staining was performed on FFPE HCC specimens using the antibody panel and experimental conditions described in the main text. Whole-slide images were acquired and managed using Phenochart (v2.2.0). TLS regions were identified on matched H&E sections and manually annotated, and TLS maturation stages were assigned according to predefined histologic criteria described in the main text. TLS annotations were subsequently transferred to the corresponding mIF images for quantitative analysis.

Cell segmentation and marker-based phenotyping were performed using vendor-provided analysis software (inForm). Quantification was restricted to cells located within annotated TLS regions. For each TLS annotation (individual TLS region), the Treg proportion was calculated as the number of CD4⁺FOXP3⁺ cells divided by the total number of cells within that TLS region, and the cDC2 proportion was calculated analogously using CD11c⁺CD1c⁺ cells. This proportion-based normalization was used to minimize confounding by TLS size.

For case-level analyses, TLS-localized Treg and cDC2 proportions were summarized across TLS regions within each case using an unweighted arithmetic mean, assigning equal weight to each TLS region and reducing disproportionate influence of larger TLS regions. This yielded one Treg and one cDC2 value per case for downstream modeling.

An mIF-based TLS RiskScore was calculated by applying the prespecified fixed-coefficient model derived from transcriptome-based analyses to the TLS-localized Treg and cDC2 values, without refitting. Patients were stratified into high- and low-TLS RiskScore groups using the cohort median as the cutoff. Overall survival (OS) and progression-free survival (PFS) analyses in the mIF cohort followed the same endpoint definitions and median-based stratification strategy described above. Associations between TLS maturation stage and TLS-localized Treg and cDC2 proportions were evaluated using nonparametric group comparisons and ordinal trend testing, as specified in the Statistical Analysis section.

**Supplementary figure**


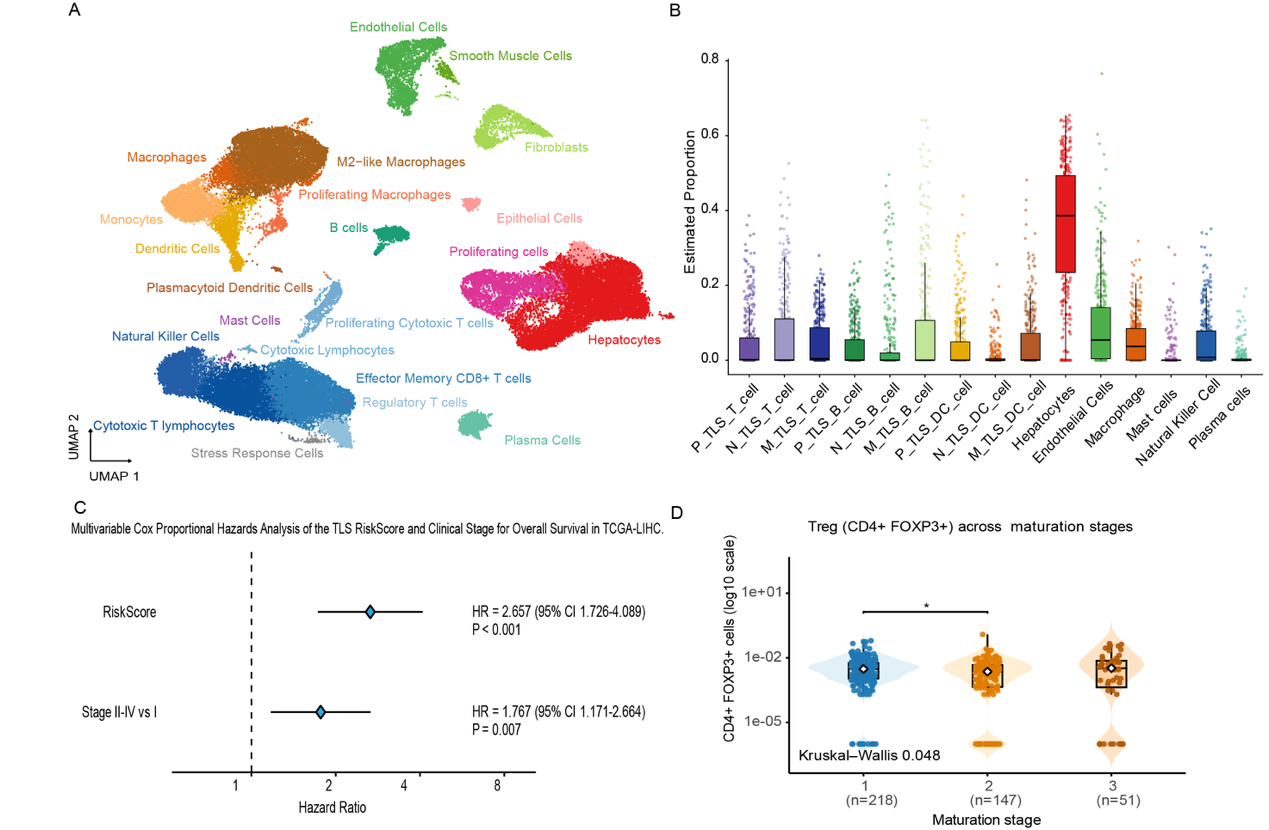


**Fig. S1** Single-cell landscape and multivariable prognostic analysis. (A) Single-cell atlas overview. UMAP projection of 59,869 high-quality single-cell transcriptomes from 10 patients, colored by annotated major cell populations as indicated. (B) Distribution of cell fractions inferred by BayesPrism across TCGA-LIHC tumors. Boxplots with overlaid individual sample points show the estimated fractions of TLS6 components and major non-TLS cell populations. Hepatocyte fractions were heterogeneous and not uniformly dominant, whereas total TLS6 fractions were measurable in most tumors. Summary statistics are provided in the Results section. (C) Multivariable Cox analysis. Forest plot showing the TLS RiskScore as an independent predictor of overall survival (HR = 2.657, P < 0.001) after adjusting for tumor stage in TCGA-LIHC. (D) Treg (CD4⁺ FOXP3⁺) cell fractions across TLS maturation stages. Violin plots with overlaid boxplots show the distribution of Treg proportions across three TLS maturation stages (n = 218, 147, and 51, respectively). Statistical significance was assessed using the Kruskal–Wallis test.

**Supplementary table**

**Table1**

| **First author, year (journal)** | **Country** | **Source type** | **cohort size (N)** | **TLS feature** |
| --- | --- | --- | --- | --- |
| Cabrita, 2020 (Nature)(1) | Melanoma | Hospital cohort | 164 | GENERIC |
| / | / | TCGA | 349 | TLS-related gene signature |
| Cinnamon, 2024 (J Hepatol)(2) | Intrahepatic cholangiocarcinoma | Hospital cohort | 118 | LOCATION |
| / | / | / | 118 | LOCATION |
| Kinker, 2023 (Gut)(3) | Pancreatic ductal adenocarcinoma | TCGA | 147 | TLS-related gene signature |
| Li, 2024 (Advanced Science)(4) | HCC | Multi-site cohort | 342 | GENERIC |
| / | / | / | 342 | GENERIC |
| / | / | / | 342 | GENERIC |
| / | / | / | 342 | GENERIC |
| / | / | / | 119 | GENERIC |
| / | / | / | 119 | GENERIC |
| / | / | / | 119 | GENERIC |
| / | / | / | 119 | GENERIC |
| / | / | / | 205 | GENERIC |
| / | / | / | 205 | GENERIC |
| / | / | / | 205 | GENERIC |
| / | / | / | 205 | GENERIC |
| Li, 2024 (Molecular Cancer)(5) | Breast cancer | TCGA | Not reported | TLS-related gene signature |
| MacFawn, 2024 (Cancer Cell)(6) | High-grade serous ovarian cancer | TCGA | Not reported | TLS-related gene signature |
| / | / | / | 501 | TLS-related gene signature |
| Ruffin, 2021 (Nat Commun)(7) | Head and Neck Squamous Cell Carcinoma | Hospital cohort | 50 | MATURITY |
| Wu, 2021 (Sci Adv)(8) | HCC | TCGA | Not reported | TLS-related gene signature |
| Calderaro, 2019 (J Hepatol)(9) | HCC | Hospital cohort | 273 | GENERIC |
| / | / | / | 273 | GENERIC |
| / | / | / | 273 | MATURITY |
| / | / | / | 217 | GENERIC |
| / | / | / | 82 | GENERIC |
| / | / | / | 214 | TLS-related gene signature |
| / | / | / | 214 | TLS-related gene signature |
| Jia, 2022 (Front Immunol)(10) | HCC | TCGA | 336 | GENERIC |
| / | / | / | 336 | GENERIC |
| Li, 2022 (Cancers)(11) | HCC | Hospital cohort | 150 | GENERIC |
| / | / | / | 150 | GENERIC |
| Li, 2021 (Front Immunol)(12) | HCC | Hospital cohort | 240 | LOCATION |
| / | / | / | 240 | LOCATION |
| Kong, 2025 (World J Oncol)(13) | HCC | TCGA | 343 | TLS-related gene signature |
| / | / | Hospital cohort | 221 | TLS-related gene signature |
| Long, 2024 (Journal for ImmunoTherapy of Cancer)(14) | HCC | TCGA | 363 | LOCATION |
| / | / | / | 363 | LOCATION |
| / | / | Multi-site cohort | 660 | LOCATION |
| / | / | / | 660 | LOCATION |
| Li, 2024 (Laboratory Investigation)(15) | HCC | Hospital cohort | 150 | GENERIC |
| / | / | / | 437 | GENERIC |
| / | / | / | 437 | GENERIC |
| / | / | / | 275 | GENERIC |
| / | / | / | 275 | GENERIC |
| Long, 2025 (Journal for ImmunoTherapy of Cancer)(16) | HCC | Multi-site cohort | 307 | GENERIC |
| / | / | / | 307 | GENERIC |
| / | / | / | 76 | GENERIC |
| / | / | / | 76 | GENERIC |
| / | / | / | 277 | GENERIC |
| / | / | / | 277 | GENERIC |

**Table 2**

| **Cluster** | **Marker** |
| --- | --- |
| **TLS-promting T cell** | CXCL13(1,3,17), CCL19(1), LTB(18), CORO1A(18), RBPJ(18), CCL4(18), ITGAE(3), IKZF1^4^, CXCL9(19), CXCL10(19), CXCL11(19), CCL5(20), CCL21(1,17), ICOS(17), CXCR5(1,3), ENTPD1(3), BATF(3), TOX(3), GZMB(2,3), IFNG(3), TNFRSF18(3), TNFSF14(17), IL21(17), BCL6(17), CXCR3(1), CCR7(1), SELL(1), TCF7(1), CD4(2), PDCD1(17), CCR5(21), CD40(21), SH2D1A(21), STAT5A(21), CD38(21), CD5(21), IL6(22), IL17A(22), IL22(22), PDPN(22), CD40LG(17), TNF(23), TBX21(24), CD69(20), PTPRC(24), MB21D1(20), RORC(25), STAT3(25), IL7(26), CXCL12(26), LTA(25). |
| **TLS-suppressive T cell** | TOX(27), TIGIT(27), HAVCR2(27), ENTPD1(27), FOXP3(17), PDCD1(23), RORC(2), GATA3(2), IL10(23), SATB1(17), TGFB1(17), IL12A(23), EBI3(23), CTLA4(28), IL2RA(25). |
| **TLS-promting B cell** | ADAM28(29), AICDA(25), BANK1(6), BCL2A1(3), BCL6(1), CCL19(1), CCL21(1), CCR1(7), CCR7(1), CD180(6), CD19(7), CD22(30), CD27(7), CD37(7), CD38(7), CD40(1), CD40LG(31), CD69(32), CD72(7), CD79A(29), CD79B(3), CD80(33), CD83(1), CD86(1), CR2(25), CXCL12(25), CXCL13(19), CCL21(1), CXCR4(25), CXCR5(19), FAS(17), FCER2(31), FCRL2(6), FCRL5(29), GFI1(21), ICAM1(7), ICOSLG(31), IKZF1(27), IL21(25), IL21R(3), IL4, IL7, IRF4(21), LTA(33), LTB(33), LTBR(25), MKI67(33), MS4A1(19), MYC(3), MZB1(3), NR4A2(27), PAX5(34), POU2AF1(6), SDC1(21), SELL(1), SEMA4A(7), SSR4(29), TNF(31), TNFRSF13C(34), TNFRSF17(21), TNFSF13(31), TNFSF13B(25), TNFSF14(25), TRAF6(21), XBP1(29). |
| **TLS-suppressive B cell** | IL10(31), PDCD1(31), TGFB1(23), IL12A(23), EBI3(23), CD274(33), CD19(33), CD22(30), CD5(35), CD27(35), CD38(35). |
| **TLS-promting DC cell** | BATF3(31), CCL19(25), CCL21(25), CD1C(36), CD40(21), CD80(31), CD83(25), CD86(24), CCR7(25), CR2(6), CXCL12(35), CXCL13(36), CXCR4(32), DCSTAMP(6), HLA-DRA(36), ICAM1(31), IL7(35), IL17A(35), ITGAX(25), LAMP3(1), LTA(31), LTB(23), TMEM173(23), TNF(31), TNFSF14(31). |
| **TLS-suppressive DC cell** | ACAT1(32), CD274(25), IDO1(32), PDCD1LG2(36), HAVCR2(36). |

**Reference**

1. Cabrita R, Lauss M, Sanna A, Donia M, Skaarup Larsen M, Mitra S, Johansson I, Phung B, Harbst K, Vallon-Christersson J, et al. Tertiary lymphoid structures improve immunotherapy and survival in melanoma. *Nature* (2020) 577:561–565. doi: 10.1038/s41586-019-1914-8

2. Cinnamon E, Stein I, Zino E, Rabinovich S, Shovman Y, Schlesinger Y, Salame T-M, Reich-Zeliger S, Albrecht T, Roessler S, et al. RORc-expressing immune cells negatively regulate tertiary lymphoid structure formation and support their pro-tumorigenic functions. *Journal of Hepatology* (2024)S0168827824027697. doi: 10.1016/j.jhep.2024.12.015

3. Kinker GS, Vitiello GAF, Diniz AB, Cabral-Piccin MP, Pereira PHB, Carvalho MLR, Ferreira WAS, Chaves AS, Rondinelli A, Gusmão AF, et al. Mature tertiary lymphoid structures are key niches of tumour-specific immune responses in pancreatic ductal adenocarcinomas. *Gut* (2023) 72:1927–1941. doi: 10.1136/gutjnl-2022-328697

4. Li J, Zhang L, Xing H, Geng Y, Lv S, Luo X, He W, Fu Z, Li G, Hu B, et al. The Absence of Intra‐Tumoral Tertiary Lymphoid Structures is Associated with a Worse Prognosis and mTOR Signaling Activation in Hepatocellular Carcinoma with Liver Transplantation: A Multicenter Retrospective Study. *Advanced Science* (2024) 11:2309348. doi: 10.1002/advs.202309348

5. Li S, Zhang N, Zhang H, Yang Z, Cheng Q, Wei K, Zhou M, Huang C. Deciphering the role of LGALS2: insights into tertiary lymphoid structure-associated dendritic cell activation and immunotherapeutic potential in breast cancer patients. *Mol Cancer* (2024) 23:216. doi: 10.1186/s12943-024-02126-4

6. MacFawn IP, Magnon G, Gorecki G, Kunning S, Rashid R, Kaiza ME, Atiya H, Ruffin AT, Taylor S, Soong TR, et al. The activity of tertiary lymphoid structures in high grade serous ovarian cancer is governed by site, stroma, and cellular interactions. *Cancer Cell* (2024) 42:1864-1881.e5. doi: 10.1016/j.ccell.2024.09.007

7. Ruffin AT, Cillo AR, Tabib T, Liu A, Onkar S, Kunning SR, Lampenfeld C, Atiya HI, Abecassis I, Kürten CHL, et al. B cell signatures and tertiary lymphoid structures contribute to outcome in head and neck squamous cell carcinoma. *Nat Commun* (2021) 12:3349. doi: 10.1038/s41467-021-23355-x

8. Wu R, Guo W, Qiu X, Wang S, Sui C, Lian Q, Wu J, Shan Y, Yang Z, Yang S, et al. Comprehensive analysis of spatial architecture in primary liver cancer. *SCIENCE ADVANCES* (2021)

9. Calderaro J, Petitprez F, Becht E, Laurent A, Hirsch TZ, Rousseau B, Luciani A, Amaddeo G, Derman J, Charpy C, et al. Intra-tumoral tertiary lymphoid structures are associated with a low risk of early recurrence of hepatocellular carcinoma. *Journal of Hepatology* (2019) 70:58–65. doi: 10.1016/j.jhep.2018.09.003

10. Jia W, Yao Q, Wang Y, Mao Z, Zhang T, Li J, Nie Y, Lei X, Shi W, Song W. Protective effect of tertiary lymphoid structures against hepatocellular carcinoma: New findings from a genetic perspective. *Front Immunol* (2022) 13:1007426. doi: 10.3389/fimmu.2022.1007426

11. Li J, Nie Y, Jia W, Wu W, Song W, Li Y. Effect of Tertiary Lymphoid Structures on Prognosis of Patients with Hepatocellular Carcinoma and Preliminary Exploration of Its Formation Mechanism. *Cancers* (2022) 14:5157. doi: 10.3390/cancers14205157

12. Li H, Liu H, Fu H, Li J, Xu L, Wang G, Wu H. Peritumoral Tertiary Lymphoid Structures Correlate With Protective Immunity and Improved Prognosis in Patients With Hepatocellular Carcinoma. *Front Immunol* (2021) 12:648812. doi: 10.3389/fimmu.2021.648812

13. Kong XY, Li XH, Qiu XL, Ma MY, Liu JH, Wang ZC, Meng ZH, Ji SW. A Tertiary Lymphoid Structure-Related Gene Signature Predicts Prognosis and Treatment Response in Hepatocellular Carcinoma. *World J Oncol* (2025) 16:587–608. doi: 10.14740/wjon2646

14. Long S, Li M, Chen J, Zhong L, Abudulimu A, Zhou L, Liu W, Pan D, Dai G, Fu K, et al. Spatial patterns and MRI-based radiomic prediction of high peritumoral tertiary lymphoid structure density in hepatocellular carcinoma: a multicenter study. *J Immunother Cancer* (2024) 12:e009879. doi: 10.1136/jitc-2024-009879

15. Li J, Xu H, Han J, Sun P, Zhang X, Wang H, Bian T, Xu Q, Ji J, Huang J. Lymphocyte Function in Tertiary Lymphoid Structures Predicts Hepatocellular Carcinoma Outcome. *Laboratory Investigation* (2024) 104:102144. doi: 10.1016/j.labinv.2024.102144

16. Long S, Li M, Chen J, Zhong L, Dai G, Pan D, Liu W, Yi F, Ruan Y, Zou B, et al. Transfer learning radiomic model predicts intratumoral tertiary lymphoid structures in hepatocellular carcinoma: a multicenter study. *J Immunother Cancer* (2025) 13:e011126. doi: 10.1136/jitc-2024-011126

17. Chaurio RA, Anadon CM, Lee Costich T, Payne KK, Biswas S, Harro CM, Moran C, Ortiz AC, Cortina C, Rigolizzo KE, et al. TGF-β-mediated silencing of genomic organizer SATB1 promotes Tfh cell differentiation and formation of intra-tumoral tertiary lymphoid structures. *Immunity* (2022) 55:115-128.e9. doi: 10.1016/j.immuni.2021.12.007

18. Lin J, Jiang S, Chen B, Du Y, Qin C, Song Y, Peng Y, Ding M, Wu J, Lin Y, et al. Tertiary Lymphoid Structures are Linked to Enhanced Antitumor Immunity and Better Prognosis in Muscle‐Invasive Bladder Cancer. *Advanced Science* (2024)2410998. doi: 10.1002/advs.202410998

19. Schumacher TN, Thommen DS. Tertiary lymphoid structures in cancer. *Science* (2022) 375:eabf9419. doi: 10.1126/science.abf9419

20. Zhao R, Zhang J, Ma J, Qu Y, Yang Z, Yin Z, Li F, Dong Z, Sun Q, Zhu S, et al. cGAS-activated endothelial cell–T cell cross-talk initiates tertiary lymphoid structure formation. *Sci Immunol* (2024) 9:eadk2612. doi: 10.1126/sciimmunol.adk2612

21. Jiang Q, Tian C, Wu H, Min L, Chen H, Chen L, Liu F, Sun Y, Department of General Surgery, Zhongshan Hospital, Fudan University, Shanghai 200032, China, Cancer Center, Zhongshan Hospital, Fudan University, Shanghai 200032, China, et al. Tertiary lymphoid structure patterns predicted anti-PD1 therapeutic responses in gastric cancer. *Chinese Journal of Cancer Research* (2022) 34:365–382. doi: 10.21147/j.issn.1000-9604.2022.04.05

22. Jia W, Zhang T, Yao Q, Li J, Nie Y, Lei X, Mao Z, Wang Y, Shi W, Song W. Tertiary Lymphatic Structures in Primary Hepatic Carcinoma: Controversy Cannot Overshadow Hope. *Front Immunol* (2022) 13:870458. doi: 10.3389/fimmu.2022.870458

23. Zhang Y, Xu M, Ren Y, Ba Y, Liu S, Zuo A, Xu H, Weng S, Han X, Liu Z. Tertiary lymphoid structural heterogeneity determines tumour immunity and prospects for clinical application. *Mol Cancer* (2024) 23:75. doi: 10.1186/s12943-024-01980-6

24. Zhao H, Wang H, Zhou Q, Ren X. Insights into tertiary lymphoid structures in the solid tumor microenvironment: anti-tumor mechanism, functional regulation, and immunotherapeutic strategies. *Cancer Biol Med* (2021) 18:981–991. doi: 10.20892/j.issn.2095-3941.2021.0029

25. Zhao L, Jin S, Wang S, Zhang Z, Wang X, Chen Z, Wang X, Huang S, Zhang D, Wu H. Tertiary lymphoid structures in diseases: immune mechanisms and therapeutic advances. *Sig Transduct Target Ther* (2024) 9:225. doi: 10.1038/s41392-024-01947-5

26. Wu X, Huang Q, Chen X, Zhang B, Liang J, Zhang B. B cells and tertiary lymphoid structures in tumors: immunity cycle, clinical impact, and therapeutic applications. *Theranostics* (2025) 15:605–631. doi: 10.7150/thno.105423

27. Lin J, Jiang S, Chen B, Du Y, Qin C, Song Y, Peng Y, Ding M, Wu J, Lin Y, et al. Tertiary Lymphoid Structures are Linked to Enhanced Antitumor Immunity and Better Prognosis in Muscle‐Invasive Bladder Cancer. *Advanced Science* (2025) 12:2410998. doi: 10.1002/advs.202410998

28. Schumacher TN, Thommen DS. Tertiary lymphoid structures in cancer. *Science* (2022) 375:eabf9419. doi: 10.1126/science.abf9419

29. Meylan M, Petitprez F, Becht E, Bougoüin A, Pupier G, Calvez A, Giglioli I, Verkarre V, Lacroix G, Verneau J, et al. Tertiary lymphoid structures generate and propagate anti-tumor antibody-producing plasma cells in renal cell cancer. *Immunity* (2022) 55:527-541.e5. doi: 10.1016/j.immuni.2022.02.001

30. Wu Z, Zhou J, Xiao Y, Ming J, Zhou J, Dong F, Zhou X, Xu Z, Zhao X, Lei P, et al. CD20+CD22+ADAM28+ B Cells in Tertiary Lymphoid Structures Promote Immunotherapy Response. *Front Immunol* (2022) 13:865596. doi: 10.3389/fimmu.2022.865596

31. Teillaud J-L, Houel A, Panouillot M, Riffard C, Dieu-Nosjean M-C. Tertiary lymphoid structures in anticancer immunity. *Nat Rev Cancer* (2024) 24:629–646. doi: 10.1038/s41568-024-00728-0

32. Jiao M, Guo Y, Zhang H, Wen H, Chen P, Wang Z, Yu B, Zhuma K, Zhang Y, Qie J, et al. ACAT1 regulates tertiary lymphoid structures and correlates with immunotherapy response in non–small cell lung cancer. *Journal of Clinical Investigation* (2025) 135:e181517. doi: 10.1172/JCI181517

33. Fridman WH, Meylan M, Petitprez F, Sun C-M, Italiano A, Sautès-Fridman C. B cells and tertiary lymphoid structures as determinants of tumour immune contexture and clinical outcome. *Nat Rev Clin Oncol* (2022) 19:441–457. doi: 10.1038/s41571-022-00619-z

34. Chung S-Y, Yeh Y-C, Huang C-J, Chiang N-J, Hsu DS-S, Chan M-H, Lu M-L, Hsu T-S, Hung Y-P, Yeh C-N, et al. Comparative impact of tertiary lymphoid structures and tumor-infiltrating lymphocytes in cholangiocarcinoma. *J Immunother Cancer* (2025) 13:e010173. doi: 10.1136/jitc-2024-010173

35. Wu X, Huang Q, Chen X, Zhang B, Liang J, Zhang B. B cells and tertiary lymphoid structures in tumors: immunity cycle, clinical impact, and therapeutic applications. *Theranostics* (2025) 15:605–631. doi: 10.7150/thno.105423

36. Reste M, Ajazi K, Sayi-Yazgan A, Jankovic R, Bufan B, Brandau S, Bækkevold ES, Petitprez F, Lindstedt M, Adema GJ, et al. The role of dendritic cells in tertiary lymphoid structures: implications in cancer and autoimmune diseases. *Front Immunol* (2024) 15: doi: 10.3389/fimmu.2024.1439413
